# ECM-transmitted shear stress induces apoptotic cell extrusion in early breast gland development

**DOI:** 10.3389/fcell.2022.947430

**Published:** 2022-08-29

**Authors:** F. Friedland, S. Babu, R. Springer, J. Konrad, Y. Herfs, S. Gerlach, J. Gehlen, H.-J. Krause, L. De Laporte, R. Merkel, E. Noetzel

**Affiliations:** ^1^ Institute of Biological Information Processing 2 (IBI-2): Mechanobiology, Forschungszentrum Jülich, Jülich, Germany; ^2^ DWI-Leibniz Institute for Interactive Materials, Aachen, Germany; ^3^ Institute of Technical and Macromolecular Chemistry (ITMC), Polymeric Biomaterials, RWTH University Aachen, Aachen, Germany; ^4^ Institute of Biological Information Processing 3 (IBI-3): Bioelectronics, Forschungszentrum Jülich, Jülich, Germany; ^5^ Advanced Materials for Biomedicine (AMB), Institute of Applied Medical Engineering (AME), University Hospital RWTH Aachen, Center for Biohybrid Medical Systems (CMBS), Aachen, Germany

**Keywords:** epithelial mechanobiology, shear strain, cellular mechanotransduction, multicellular spheroids, apoptosis, cell extrusion, basement membrane

## Abstract

Epithelial cells of human breast glands are exposed to various mechanical ECM stresses that regulate tissue development and homeostasis. Mechanoadaptation of breast gland tissue to ECM-transmitted shear stress remained poorly investigated due to the lack of valid experimental approaches. Therefore, we created a magnetic shear strain device that enabled, for the first time, to analyze the instant shear strain response of human breast gland cells. MCF10A-derived breast acini with basement membranes (BM) of defined maturation state and basoapical polarization were used to resemble breast gland morphogenesis *in vitro*. The novel biophysical tool was used to apply cyclic shear strain with defined amplitudes (≤15%, 0.2 Hz) over 22 h on living spheroids embedded in an ultrasoft matrix (<60 Pa). We demonstrated that breast spheroids gain resistance to shear strain, which increased with BM maturation and basoapical polarization. Most intriguingly, poorly developed spheroids were prone to cyclic strain-induced extrusion of apoptotic cells from the spheroid body. In contrast, matured spheroids were insensitive to this mechanoresponse—indicating changing mechanosensing or mechanotransduction mechanisms during breast tissue morphogenesis. Together, we introduced a versatile tool to study cyclic shear stress responses of 3D cell culture models. It can be used to strain, in principle, all kinds of cell clusters, even those that grow only in ultrasoft hydrogels. We believe that this approach opens new doors to gain new insights into dynamic shear strain-induced mechanobiological regulation circuits between cells and their ECM.

## 1 Introduction

Breast gland tissue is continuously exposed to various mechanical cues that regulate epithelial tissue development, homeostasis and tumor progression. During adaptation to changing physiological demands, *i.e.*, puberty, lactation and involution, breast epithelial cells are exposed to static fluid pressure, compressive stress, and radial contraction forces by myoepithelial cells. Contractile (myo-) fibroblasts that populate the connective tissue remodel the extracellular matrix (ECM) and generate additional dynamic mechanical stresses (Butcher et al., 2009; Northcott et al., 2018). ECM-originated tensile, compressive and shear stresses are sensed by cells via mechanoresponsive integrin receptors, which connect extracellular cell adhesion compounds, such as collagens, laminins and fibronectin (Kass et al., 2007). These integrin-based focal adhesions mechanically link the ECM to the intracellular, force-generating actomyosin network (Miyamoto et al., 1998; Belkin and Stepp, 2000; Brakebusch and Fässler, 2003). Dynamic ECM signals, such as stiffness, compression, expansion, fluid flow and solid shear stress, define the cells’ mechanical microenvironment. Cells actively sense and respond to ECM cues by intracellular mechanobiological transduction cascades, which orchestrate tissue morphogenesis (Wells, 2008; Eisenhoffer et al., 2012; Amer et al., 2021). The investigation of how tissue strain could modulate cell differentiation is a challenging task necessary to comprehend mechanobiological regulation of tissue homeostasis. Moreover, it might guide new approaches for fighting diseases linked with altered mechanical cellular microenvironments.

Various mechanobiological cell culture approaches have been developed to study the impact of compression, spatial confinement, and matrix stiffness on proliferation, migration, differentiation and gene expression of single and monolayered cells and cell spheroids (Mohammed et al., 2019). Cell stretching devices have been developed to apply defined cyclic uniaxial strain on cells and clusters on planar substrates (Niediek et al., 2012). Such cyclic strain can induce the remodeling of cell-matrix and cell-cell junctions in skin differentiation (Noethel et al., 2018). Engineered microfluidic devices simulate fluidic shear stresses in vascular systems driving endothelial differentiation of mesenchymal progenitor cells (Wang et al., 2005; Paddillaya et al., 2019). In addition, both uniaxial and fluid shear strain has been shown to trigger the reorientation of cytoskeletal components and the reciprocal remodeling of the ECM by strained cells (Asanuma et al., 2003; Niediek et al., 2012; Russo et al., 2020; Vion et al., 2021). These approaches gained groundbreaking knowledge about cellular response mechanisms to extracellular mechanical stresses. However, most findings were achieved by analyzing cells that interacted with planar elastomeric surfaces. It is known that more physiological 3D matrices modulate distinct signaling cascades and cellular differentiation programs even within the same type of cell (MJ et al., 2003; Kapałczyńska et al., 2016; Jensen and Teng, 2020). Therefore, the three-dimensionality of the cellular microenvironment has to be taken into account to understand ECM-cell interactions fully. Thus, natural and synthetic hydrogels with adjustable stiffness were utilized to study tissue-specific cell niches (Raeber et al., 2005; Taubenberger et al., 2019). For example, NIH/3T3 fibroblasts embedded in gelatin methacrylate gels of varying stiffness have been uniaxially stretched to probe their response to extreme strain (Li et al., 2016).

Natural EHS-based hydrogels are commonly used to generate basoapically polarized breast spheroids of the normal-like MCF10A cell line. These spheroids mirror many features of healthy breast gland tissue with apical lumen and basement membrane (BM) formation (Debnath et al., 2003; Gaiko-Shcherbak et al., 2015). This BM scaffold separates cells from the surrounding ECM and is a hallmark of homeostatic epithelial tissues (Khalilgharibi and Mao, 2021). Moreover, the BM provides significant mechanical stability for breast spheroids (Gaiko-Shcherbak et al., 2015; Fabris et al., 2018; Khalilgharibi and Mao, 2021). A polarization loss can be triggered by tumor-like ECM stiffening and oncogenic EGF signaling in these breast spheroids. The resulting invasive behavior is accompanied by epithelial to mesenchymal transition-like reorganization of the actin cytoskeleton that finally contributes to cell force-driven BM disruption (Gaiko-Shcherbak et al., 2015; Gaiko-Shcherbak et al., 2021). Therefore, mechanoresponsive MCF10A breast spheroids are well-appreciated and versatile *in vitro* models to study normal epithelial breast gland development and breast tumor progression (Debnath et al., 2002; Debnath et al., 2003; Vitolo et al., 2009; Qu et al., 2015; Puleo and Polyak, 2021).

*In vivo*, the mammary epithelium is exposed to solid tissue strain. For example, Haake and Scurr measured the overall motion of the female breast during running and reported strain amplitudes of approximately 50% or more (Haake and Scurr, 2011). This deformation of the breast epithelium surrounding connective tissue is best resembled by shear strain in an elastic material. How such shear stresses could modulate tissue development and homeostasis remains elusive. Notably, missing suitable biophysical tools limited the investigation of solid shear strain as a regulator of tissue homeostasis. To accommodate this lack, we designed a novel magnetic spring device that allowed, for the first time, to exert defined cyclic shear stress amplitudes on breast spheroids that were embedded in ultrasoft natural hydrogels. This new device combined high-resolution live-cell imaging with fine-tunable cyclic strain to investigate epithelial cell response to nature-like mechanical strain. We carefully characterized the reproducibility and robustness of long-term cyclic strain of ultrasoft hydrogels. Importantly, we found that strain-induced apoptotic cell extrusion is dependent on the developmental stage of MCF10A breast spheroids. We are confident that our new tool will enable valuable insights into strain-induced mechanotransductive regulation circuits in all types of cells that grow in natural and synthetic hydrogels.

## 2 Materials and methods

### 2.1 Preparation of cell culture dishes and magnetic sample lids

Samples were prepared in 35 mm Ø cell culture dishes with an inner well of 18 mm Ø. A 22 mm Ø type 0 cover glass was attached with silicone elastomer prepared according to the manufacturer’s specifications (Sylgard 184, Dow Corning, Midland, MI, United States). Magnetic sample lids were fabricated by attaching a 1 mm cubic NdFeB (N45, Ni-Cu-Ni coating, Webcraft, Gottmadingen, Germany) magnet on top of a 13 mm Ø type 1.5 cover glass or 12 mm Ø titanium mesh (type TI00-MS-000100, Goodfellow, Hamburg, Germany) using silicone elastomer. The magnet was embedded entirely in silicone to avoid corrosion and possible leakage of toxic components of the magnet’s coating. Magnetic lids made from cover glasses were sterilized in a plasma cleaner. Magnetic lids made from titanium meshes were sterilized with isopropanol. Titanium meshes were used for magnetic sample lids for long-term cyclic strain experiments to ensure sufficient nutrition and media diffusion. For all other experiments, magnetic sample lids were made from cover glasses.

### 2.2 Cell maintenance

MCF10A cells were purchased from ATCC (Manassas, VA, United States) and maintained in culture dishes under standard culture conditions (37°C, 5% CO_2_) in DMEM/F12 growth medium (ThermoFisher Scientific, Waltham, MA, United States) containing 5% horse serum (ThermoFisher Scientific), 0.5 μg/ml hydrocortisone, 100 ng/ml cholera toxin, 20 ng/ml EGF, 10 μg/ml insulin (all Sigma Aldrich, St. Louis, MO, United States), 100 U/mL penicillin and 100 μg/ml streptomycin (ThermoFisher Scientific). For MCF10A 3D morphogenesis, a DMEM/F12 assay medium was applied (see below). In addition, an MCF10A cell variant transduced with an RFP-LifeAct construct (IBIDI, Munich, Germany) was used to visualize the actin cytoskeleton during live-cell imaging experiments.

### 2.3 Cultivation of MCF10A spheroids

Single MCF10A cells were seeded between two layers of growth factor reduced EHS-gel (Geltrex, Life Technologies) with embedded fluorescent microspheres (Invitrogen, Fluospheres™ carboxylate-modified, 0.2 µm, dark red fluorescent, 4.6 × 10^12^ particles/mL). To prepare EHS-gel-marker bead mixtures, beads were centrifuged at 13.000 g and washed with ice-cold PBS twice before being resuspended in PBS and mixed with the hydrogel (1:50, v/v). The mixture was kept on ice during the entire sample preparation. First, a bed of 100 µL EHS-gel was prepared and cells were placed on top as described in previous studies as “on top” cultivation (Debnath et al., 2002; Gaiko-Shcherbak et al., 2015). After discarding excess medium and evaporation of remaining liquid for 10 min, the second layer of 50 µL EHS-gel was placed on top of the gel bed and left to solidify at 37°C before assay medium was added. Samples were kept at 37°C in a humidified environment of 5% CO_2_ and 95% air. Assay medium was changed every 3–4 days. Assay medium: DMEM/F12 (ThermoFisher Scientific) containing 2% horse serum (Life Technologies), 5 ng/ml epidermal growth factor (day 1–9) (EGF, Sigma-Aldrich), 0.5 μg/ml hydrocortisone (Sigma-Aldrich), 100 ng/ml cholera toxin (Sigma-Aldrich), 10 μg/ml, 100 U/mL penicillin and 100 μg/ml streptomycin (Sigma-Aldrich).

### 2.4 Sample preparation for shear strain experiments

MCF10A spheroids were prepared 1 h before the start of experiments by first discarding the assay medium and letting the excess liquid evaporate for 10 min without drying out of the hydrogel. Samples were placed in a magnetic positioner (see Figure 2C) before adding 50 µL of fresh hydrogel to attach a magnetic sample lid on top of the gel. After letting the gel solidify, assay medium (EGF-free) containing 25 mM HEPES (2-[4-(2-Hydroxyethyl)piperazin-1-yl]ethane-1-sulfonic acid, ThermoFisher Scientific) was added and the dishes were sealed with Parafilm. For immunocytochemical treatment, shear stress samples were fixated with 2% formaldehyde (Merck Millipore, Billerica, MA, United States). and 1% glutaraldehyde (Polysciences, Warrington, PA, United States) in cytoskeleton-buffer (CB: 5 mM ethylene glycol-bis(β-aminoethyl ether)-N,N,N′,N′-tetraacetic acid, 5 mM glucose, 10 mM 2-(N-Morpholino)ethansulfonsäure, 5 mM MgCl_2_, 150 mM NaCl, 1 g/L streptomycin; all Sigma Aldrich) for 20 min at room temperature (RT) and washed with 30 mM glycine-CB. Quenching of the EHS-matrix was performed with 1% NaBH_4_ (freshly prepared in CB) (Merck Millipore, Billerica, MA, United States).

### 2.5 MCF10A spheroid transfer and fixation

ECM-embedded MCF10A spheroids were cultivated as described in 2.3 and incubated in 2 ml ice-cold Cell Recovery Solution (BD Biosciences, San Jose, CA, United States) for 30 min (4°C) to fluidize the EHS matrix. After mechanical disruption of the matrix with EGF-free medium, individual acini were picked under a stereomicroscope and placed into fresh EGF-free medium. The spheroid solution was transferred onto a cover glass coated with EHS-gel by incubation for 2 h with 2 μg/ml EHS-PBS solution. After letting the spheroids adhere to the cover glass for 30 min (37°C, 5% CO_2_), they were fixated with 3.7% formaldehyde-CB for 10 min at RT and stored at 4°C in CB buffer.

### 2.6 Immunocytochemistry

Permeabilization was performed with 1% Triton X-100-CB (Sigma-Aldrich) solution for 10 min at RT. Unspecific antibody binding was blocked with blocking buffer (0.1% BSA, 0.2% Triton X-100, 0.05% Tween 20, in CB; Sigma- Aldrich) containing 5% skim milk powder (Sigma-Aldrich), and 1% Goat Anti-Mouse IgG, F (ab')₂ fragment specific (20 μg/ml, Jackson ImmunoResearch, United Kindom) for 2 h at RT. Primary antibodies anti-type IV collagen (Abcam), anti-cleaved caspase-3 (Cell Signaling Technology) or anti-GM 130 (BD Biosciences) were diluted 1:200 in 1% blocking buffer in CB and incubated overnight at 4°C. Secondary antibodies coupled with fluorescent dyes were equally diluted and applied to samples for 45 min at RT in darkness. Before storing the samples in darkness at 4°C they were treated with Nuc Blue Fixed Cell Stain (Invitrogen) or DRAQ5™ (Cell Signaling) for 10 min and washed thrice with cold CB.

### 2.7 Image acquisition and processing

Live-cell imaging (LCI) was performed at 37°C and 5% CO_2_ (cell incubator XL, Zeiss, Germany) with an inverse confocal laser scanning microscope (LSM880 with Airyscan detector) that used a 40x LD C-Apochromat water immersion objective (NA = 1.1, Zeiss). For LCI, the Fast Airy Mode was used. For fixed samples, the Resolution vs Signal Mode was used. All images acquired using the Airy-scan detector were processed in 2D mode using the Zeiss ZEN Black software (Zeiss, Germany). Imaris (version 9.8) was used to create 3D surface renderings from z-stacks (pixel size: x = 0.12 µm; y = 0.12 µm; z = 0.1 µm) of MCF10A actin signal.

### 2.8 Displacement determination by template matching and tracking

Intensity-based image registration by means of normalized cross-correlation was used to determine microsphere displacements and the translation of image templates. All images were smoothed by a Gaussian filter with a sigma of 0.5. The first or reference image (pixel size: x = 0.18 µm, y = 0.18 µm) was divided into small square templates with side lengths of 79 pixels (samples day ≤4) or 119 pixels (samples day >4) that were searched for in the following images of the image sequence by normalized cross-correlation. The threshold for the normalized cross-correlation coefficient was set to 0.5 (default value). The difference of the template positions was the displacement.

### 2.9 Linear regression and statistical analysis

For evaluation of strain in gels and spheroids, the median value of displacement in each image plane was used as x value, with the corresponding height being the y value as basis for linear regression. Linear regression of median template displacement described in 2.5 was calculated using the ordinary least squares method with the statsmodels module in python 3. For all analyses, the two-tailed nonparametric Mann–Whitney *U*-test and two-sample Kolmogorov-Smirnov test were performed using the scipy. stats module in Python 3. The *p*-values were defined as follows: ****: *p* < 0.0001; ***: 0.0001 ≤ *p* < 0.001; **: 0.001 ≤ *p* < 0.01; *: 0.01 ≤ *p* < 0.05; n. s. *p* ≥ 0.05; Significance result of Mann-Whitney *U*-test was only indicated if Kolmogorov-Smirnov test resulted in *p*-values below 0.05 as well.

### 2.10 Rheological analysis

The shear moduli of pure and diluted (70% Geltrex, 30% DMEM/F12) hydrogels were measured on a DHR three Rheometer from TA instruments. The measurements were recorded at 37°C using a cone and plate geometry of 20 mm diameter, with a 2° cone angle and at 51 μm truncation gap. A solvent trap was used containing excess water to prevent the sample from drying out during the tests. Time sweep measurements were recorded for each sample at 0.5 Hz frequency and 0.5% strain for 30 min until the storage modulus curve reached a plateau. The time point at which the loss tangent (tan (δ)) value dropped below 0.1 was taken as the point of gelation. The storage modulus was calculated by averaging over all values after this time point.

## 3 Results

### 3.1 A magnetic LCI-device to apply solid shear stress on hydrogel-embedded spheroids

We hypothesized that naturally occurring extracellular mechanical strain could affect differentiation and homeostasis of human breast gland tissue, even in ECM of physiological stiffness (∼170 Pa) (Paszek et al., 2005). To address this question, we created a cell culture device to apply cyclic shear strain on basoapically polarizing breast cell spheroids that develop only within an ultrasoft hydrogel matrix. Our new tool had to meet several criteria to gain physiologically meaningful results: 1. Generation of defined and reproducible solid shear strain in hydrogels to model physiological conditions. 2. Compatibility with compliant physiological EHS-matrices that enable differentiation of BM-covered MCF10A cell breast spheroids. 3. Sterile long-term live-cell imaging (LCI) ability during shear strain application. Such real-time cell tracking is crucial for analyzing mechanical stress response mechanisms. Based on these experimental demands, we designed a magnetic 3D shear stress device.

**FIGURE 1:**
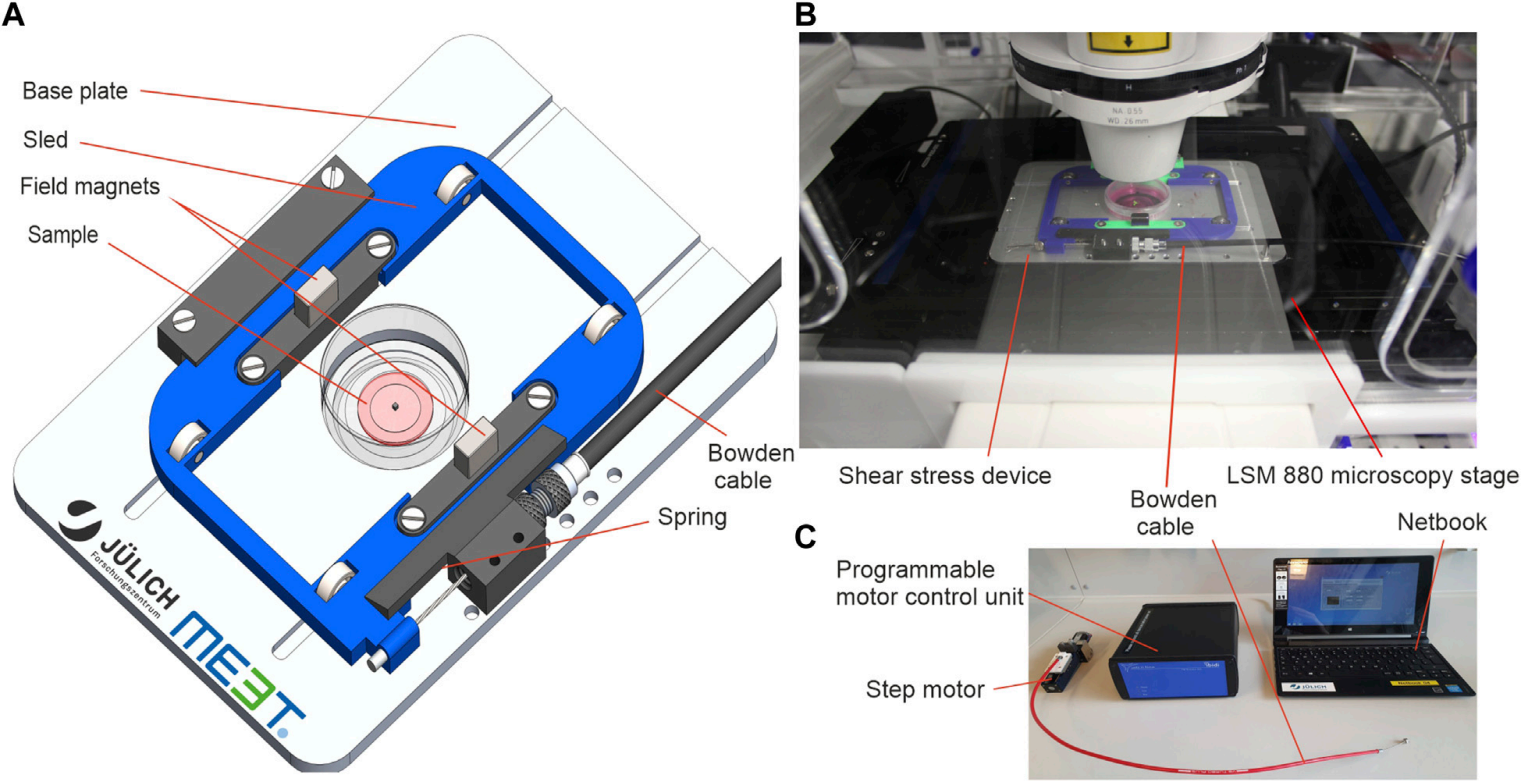
A magnetic device to apply solid shear stress on ultrasoft hydrogels. **(A)** Engineering schematic of the magnetic shear stress device. **(B)** Image of the device mounted onto the table of an inverse confocal microscope with LCI incubation unit. The step motor and the control unit are located outside the incubator. **(C)** Image of the accessory tools used to operate the device. Custom software on a standard netbook is used to program a motor control unit with a drive protocol with defined speed, amplitude, dwell time and number of repetitions. The motor control unit drives a high-precision step motor connected to the device via a Bowden cable.

**FIGURE 2:**
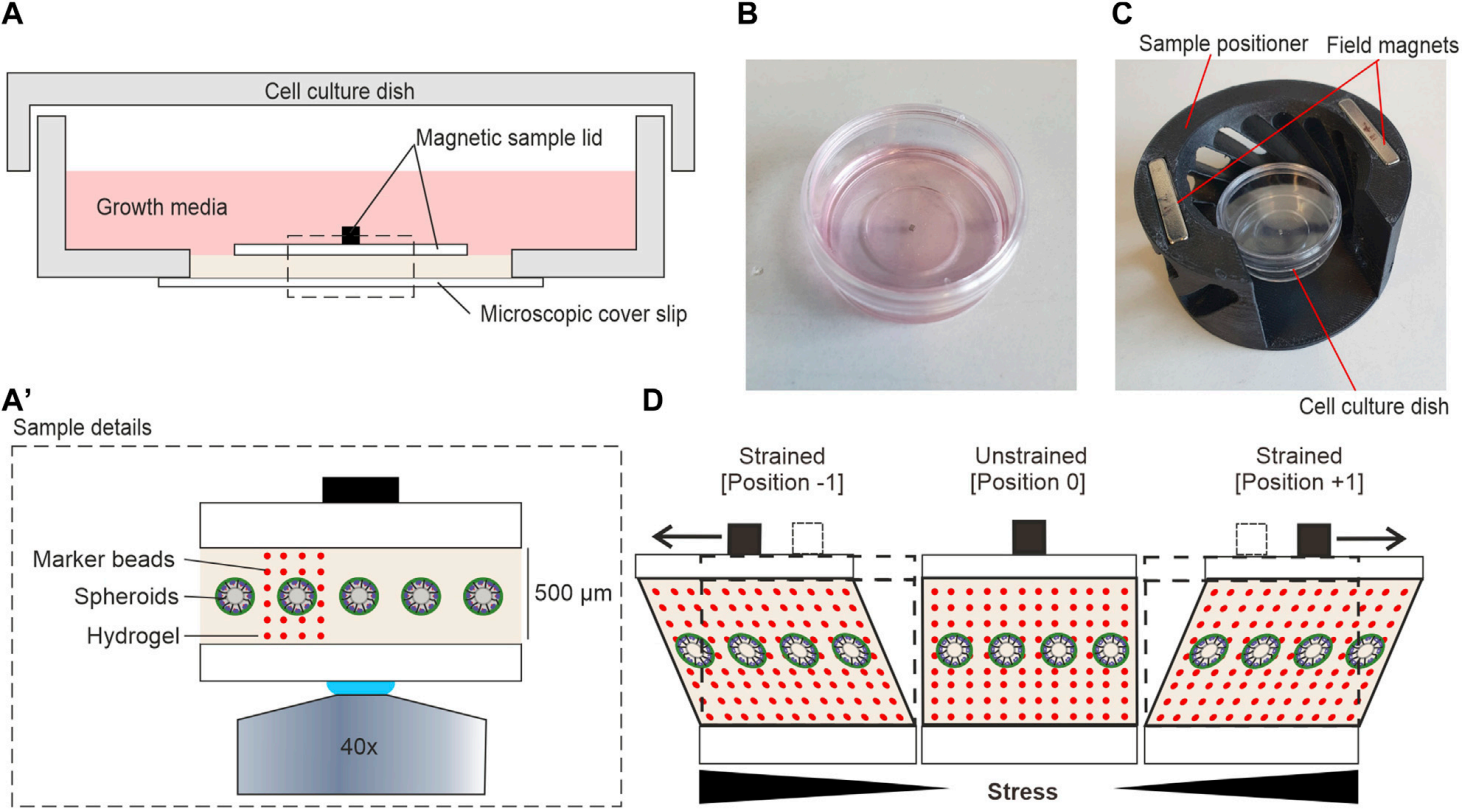
Hydrogel sample setup for shear stress application. **(A)** Detailed view on the shear strain sample within a 35 mm cell culture dish. **(A′)** Magnification of highlighted area (dashed line) in **(A) (B)** A sterile ready-to-use sample. **(C)** A sample was placed into the magnetic positioner before mounting in the magnetic field of the moving sled. **(D)** The cartoon highlights the principle of sample movement and shear stress application. Sled movement (not shown) generates a pulling force (black arrows) on the sample magnet. In strained positions, spheroids and the gel are both sheared. Local displacements at individual confocal image planes can be tracked by incorporated fluorescent marker beads (red dots).

The device consisted of a motorized moving sled with two cuboid NeFeB magnets (30 × 10 × 5 mm). In the center of the magnetic field, we placed a standard cell culture dish equipped with a magnetic coverslip to actuate a hydrogel sample (Figure 1A). The base plate was designed to fit the standard microscope table of Carl Zeiss inverted microscope bodies (Figure 1B). Due to the compact design, it can be easily adapted to other microscopes. A hole beneath the sample’s base plate enabled real-time imaging during stress application with inverse confocal microscopes. Sled movement was driven by a preloaded spring and antagonistic pulley (Bowden cable). The Bowden cable was linked to a motor unit (Figure 1C) outside the LCI-incubator of the microscope. This spatial separation of sample and motor drive allowed both sample actuation in cell culture incubators and in LCI microscopes. Further, it assured optimal imaging conditions by eliminating heating and vibrations. The motor unit was connected to a simple netbook running an in-house designed software to control the amplitude of cyclic displacement of the sled by a step motor. Whenever the sled is moved, the magnet on top of the sample is pulled towards the sled’s center. This magnetic force acted like a very soft spring but without any mechanical connections that would have transmitted an additional torque onto the sample. In essence, sled motion resulted in a force applied parallel to the surface of the hydrogel sample (cf. Figures 1A, 2D).

Figure 2A illustrates the detailed composition of the sample. The contactless sample actuation allowed the preparation of samples in sealed cell culture dishes in a sterile environment before mounting in the shear strain device. This procedure simplified sample handling and reduced contamination risk. Most importantly, it allowed prolonged LCI experiments under optimal cell culture conditions. To cultivate MCF10A breast spheroids, 500 µm thick hydrogels were prepared in the center of a glass-bottom dish as described in Section 2.3. The hydrogel was sandwiched by the magnetic sample lid and the coverslip forming the bottom of the cell culture dish. Attachment of the magnetic sample lid with additional hydrogel was done in a specially fabricated magnetic positioner (Figure 2C) to ensure a perfectly centric position of the sample magnet on top of the spheroid-bearing hydrogel. EGF-free assay medium overlayed the entire sample to avoid sample drying during LCI analysis.

Figure 2D shows the principle of force application parallel to the gel surface. We found that the EHS-hydrogel sticks to the bottom and top glass surfaces by physisorption. Hence, the movement of the upper magnetic sample lid parallel to the fixed bottom glass resulted in a shear deformation of the gel. This deformation mode transforms a square into a parallelogram. Here, the in-plane displacement of objects increases with distance to the bottom. Thereby, the generated shear strain increases the in-plane lateral displacement of the gel with increased distance to the fixed bottom. Experimentally, the cyclic movement of the sled resulted in two distinct strained positions (-1 and +1) and one unstrained, centric position (0) (Figure 2D). For all experiments, fluorescent marker beads were embedded into the gel to track the amplitudes of hydrogel displacement over the entire height.

### 3.2 Quantification of shear strain in ultrasoft hydrogels

**FIGURE 3:**
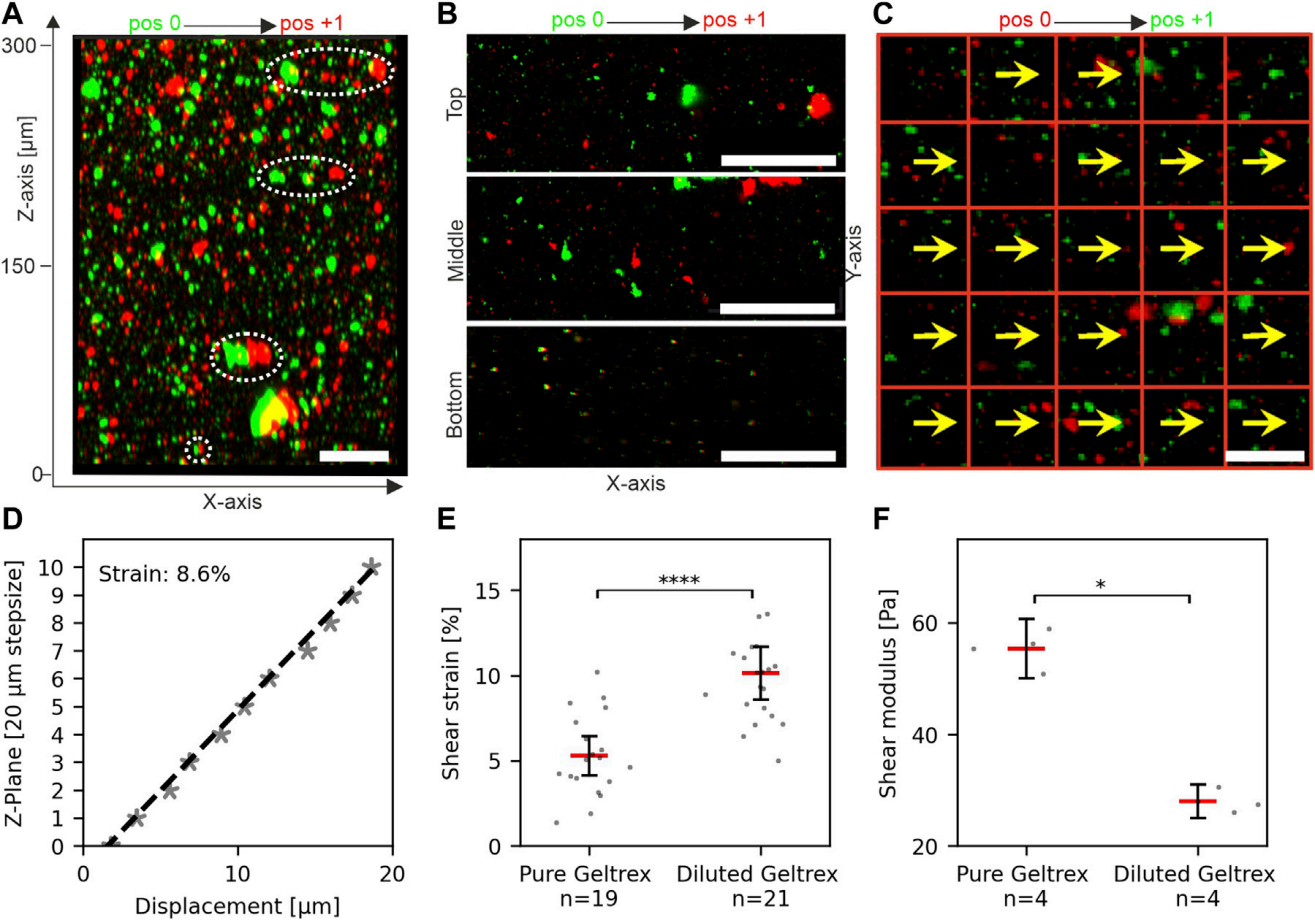
Measurement and quantification of shear strain in hydrogels. **(A)** Reconstructed image stack of fluorescent microspheres in an unstrained (pos 0, green) and strained (pos +1, red) hydrogel. White ellipses highlight the height-dependent increase of bead displacements. **(B)** Representative micrographs show bead displacements parallel to sled movement and applied force (*x*-axis). **(C)** Template displacements for an individual focus plane. Displacement vectors (DVs) (yellows arrows) are given for templates (red squares). **(D)** Linear regression (dashed line) was used to determine the applied shear strain within the gel. Each data point represents the median displacement of all detected DVs in the respective plane. Value 0 µm: indicates the first plane of the recorded image stack (absolute gel height >300 µm). The local strain was calculated from the slope of the regression line. **(E)** Scatter plots of shear strain in pure and diluted hydrogels at 5 mm sled movement, *i.e*., identical shear force. N = total number of measurements from three individual gels. The threshold for linear regression was *r* ^2^ > 0.7. **(F)** Shear moduli of pure and diluted hydrogels as determined by rheology. N = total number of measured gels. Red bars in scatter plots: mean, bars 95% confidence interval. For statistical tests, the Mann–Whitney *U*-test was used with: **** = *p* < 0.0001, * = *p* < 0.05. Scale bars: 50 µm.

Next, we tested the application of shear stress to thick hydrogels. For this, EHS-hydrogels of approx. 300 µm in height (100 µL Geltrex) were prepared and placed into the device, as described above. The displacement of embedded fluorescent microspheres was tracked at positions 0 and +1 (sled amplitude: 5 mm). Figures 3A,B show the overlay of images taken at both sled positions to visualize the increase of local displacements with height in z/x and x/y directions, respectively. The coordinate system is defined with focus position as the *z*-axis value and sled motion along the *x*-axis. A software was developed to track sample displacements in image stacks automatically. Each focal plane (z-coordinate) was analyzed separately. Details are given in Section 2.5. The representative analysis demonstrates the almost perfect parallel orientation of DV and sled motion and very little variation in DV lengths at one image plane (Figure 3C).

Together, these results indicated that the device was suitable for applying stresses to ultrasoft matrices with stiffness comparable to human breast tissue (Krouskop et al., 1998). Further, they demonstrated that the strain measurements were sensitive enough to detect changes in stiffness of ultra-soft hydrogels.

Figure 3D shows a representative plot of median displacements over multiple image planes of an entire gel. Linear regression gave a local strain of 8.6% in the gel at 5 mm sled movement. Repeated measurements at multiple areas of three independent gel samples were performed (Figure 2E). For pure hydrogel samples, a median shear strain of 5.3% was determined with a scatter as expected for biological materials. We noticed that the sample magnet was displaced ∼20 µm by a 5 mm sled movement as intended by the magnetic spring design. As sample magnet displacement is negligible compared to sled motion, the “magnetic spring” is much softer than the sample. Therefore, our setup applies defined shear stress. To test this, we diluted the hydrogel with 30% DMEM/F12 and repeated strain measurements. Matrix dilution resulted in a significant increase of median strain to 10% (increase of 92%) (Figure 2E, pure Geltrex ∼5.3%, diluted Geltrex ∼10.1%). In line with this strain increase, rheology demonstrated a significant decrease in gel stiffness to 50.6% (storage moduli: pure Geltrex ∼55 Pa and diluted Geltrex ∼28 Pa) (Figure 2F). Fully in line with expectations, the product of shear strain and storage module was constant. Thus, stress application functioned as intended; a specific sled displacement resulted in a specific tangential stress acting on the hydrogel surface.

### 3.3 Breast spheroids exhibit significant mechanical resistance to strain

**FIGURE 4:**
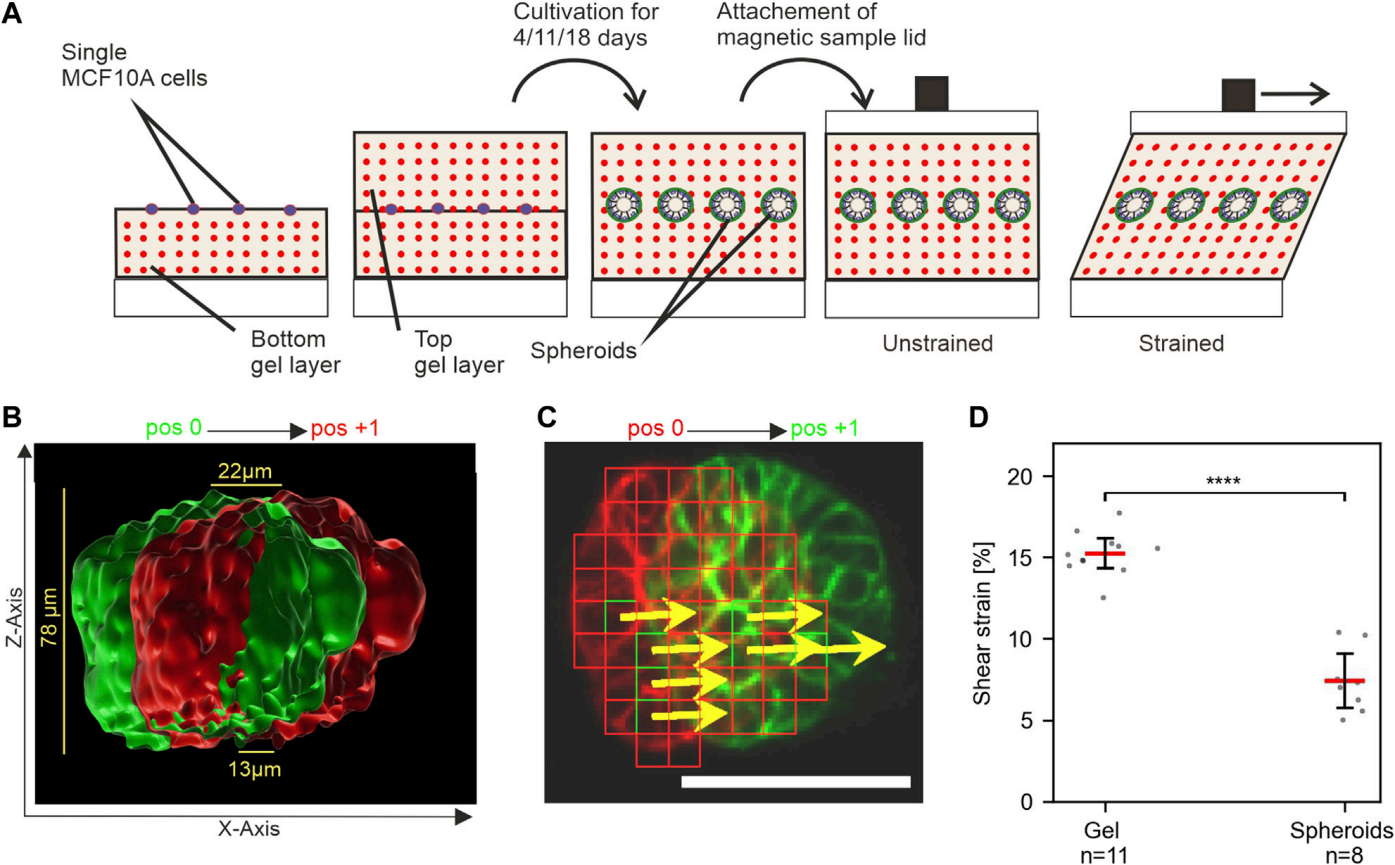
MCF10A spheroids show resistance to mechanical shear strain. **(A)** Schematic of the experimental setup to apply shear strain on hydrogel-embedded MCF-10A spheroids. Single cells are seeded between two layers of gel and cultivated upon the desired maturation stages. The magnetic lid is attached before stress application. **(B)** Surface-rendered image of the actin cytoskeleton (Liveact RFP) of a representative spheroid in unstrained (green) and strained (red) positions. The cross-section view highlights its height-dependent displacement. **(C)** Automated detection of displaced cellular actin structures for an individual focal plane. Displacement vectors (DVs) (yellows arrows) are given for templates (red squares). Scale bar: 50 µm **(D)** Measured strain amplitudes for spheroids and their surrounding gel at n positions in a single sample. Scatter plots: bars: median with 95% confidence interval. For statistical tests, Mann–Whitney *U*-test was used with: **** = *p* ≤ 0.0001, *** = *p* ≤ 0.001, * = *p* ≤ 0.05.

We aimed to develop a novel method for applying mechanical stress to 3D cell cultures to enable novel investigative approaches. For this purpose, well-described MCF10A-derived breast spheroids were chosen, since they mimic many natural breast gland epithelium features. *In vivo*, the breast gland is embedded in extremely soft tissue with a stiffness similar to EHS gels (Paszek et al., 2005). For the application of strain to MCF10A spheroids, single cells with fluorescently labeled actin cytoskeleton (LifeAct RFP-labeled) were seeded between two layers of EHS-Gels with embedded fluorescent microspheres. They were cultivated for 11 days to form breast spheroids (Figure 4A). Shear stress was applied to EHS-gels with embedded spheroids by 5 mm sled displacement. Displacement of both the microspheres and actin upon strain application was imaged. As shown in Figure 4B, the displacement of actin structures increased with the gel height, confirming qualitatively that the embedded spheroids are indeed sheared upon stress application. For quantitative analysis, strain amplitudes in spheroids were determined by template matching of actin signals in images taken in the strained and unstrained state. Only stacks where linear regression (cf. Figure 3) resulted in an *r* ^2^ above 0.7 were used. We noticed higher average strain values compared to our previous results, which we attributed to the different geometrical setup of the samples consisting of two gel layers. More intriguingly, however, was the significantly lower strain within the spheroids compared to the surrounding matrix. This result implied an inherent resistance of MCF10A spheroids to mechanical stress transmitted by the sheared ECM. We were intrigued by what could be a defining factor for this resistance and theorized that the developmental status of the spheroids and thus the maturation status of the BM played an elementary role in their mechanical resistance.

**FIGURE 5:**
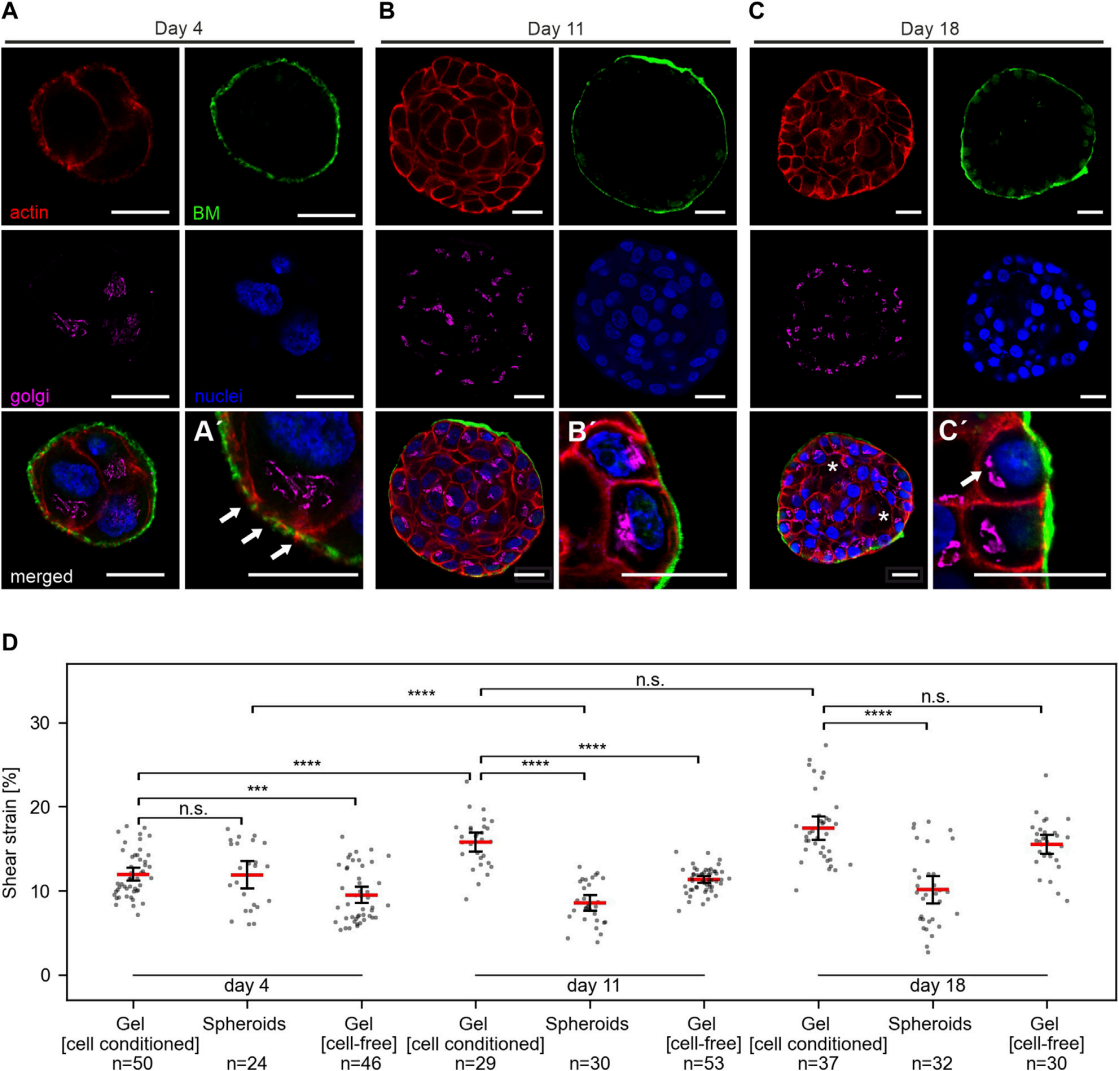
Breast spheroids gain shear strain resistance with advanced basoapical polarization. **(A–C)** Representative micrographs indicate the basoapical polarization process of MCF10A spheroids. Spheroids were cultivated over 4–18 days, as described in Figure 3A. Samples were fixed and IF-stained. Cross-sections at the equatorial plane demonstrate BM formation (collagen type IV, green), the cellular orientation of the Golgi apparatus (GM130, magenta), the actin cytoskeleton (F-actin, red) and cell nuclei (DRAQ-5, blue). Partial lumen clearance was evident on day 18 (white asterisk). Antibody control staining for collagen type IV is provided in Supplementary Figure 1F. **(A′)** Magnification highlights BM formation and thin collagen penetrating actin protrusions. **(B′)** Magnification highlights BM maturation and partial apical Golgi orientation. **(C′)** Magnification highlights a high degree of basoapically polarization. **(D)** Scatter plots summarize the applied shear strain on hydrogels (pure) MCF-10A spheroids with different maturation stages, hydrogels populated by spheroids (cell-conditioned) and cell-free hydrogels. Strain was measured at 5 mm sled displacement. N = total number of measured positions for three individual gels. Scatter plots: bars: median with 95% confidence interval.

Figure 5 A-C shows representative images of MCF10A developing spheroids cultivated in the gel between two EHS-hydrogel layers for 4, 11 or 18 days. On day 4 of development, spheroids appeared poorly developed with unorganized cell clusters and thin BM scaffold. This thin BM was frequently penetrated by actin protrusions (Figure 5A', white arrows), as reported previously (Eschenbruch et al., 2021). Basoapical cell polarization was reached partially at day 11 and fully at day 18, at which partial lumen clearance was regularly observed. Previously, our group described similar morphogenesis for MCF10A breast spheroids with gradual BM-maturation of a polarized basal cell layer and apical lumen formation (Gaiko-Shcherbak et al., 2015). These results showed that the adapted cell culture conditions and sample design for strain experiments did not affect the basoapical polarization schedule of MCF10A breast cell spheroids.

We applied shear stress to samples of MCF10A spheroids in EHS-gel cultivated for 4, 11 or 18 days. For each condition, three independent samples were analyzed. Strain measurements for spheroids, cell-conditioned gels and cell-free gels are shown in Figure 5D. Interestingly, no significant difference between strain in the spheroids and the surrounding gel was observed at day 4. However, strongly significant differences were measured at day 11 and day 18. Notable was also an increase in gel strain throughout cultivation time, with an average strain of 12% at day 4, 15.7% at day 11 and 17.4% at day 18. In line with our previous results of higher strains in softer, diluted gels, we attributed this phenomenon to a gel softening that seemed to occur during cultivation. To test whether this gel softening was caused by cellular ECM remodeling, we also analyzed identically prepared but cell-free samples after the same cultivation time. In these cell-free gels, a similar strain increase was observed with average strain values of 9.5% at day 4, 11.3% at day 11, and 15.5% at day 18. Strain in cell-containing and cell-free gels was significantly different at day 4 and day 11, but not at day 18.

### 3.4 Cyclic shear strain triggers aberrant apoptotic cell extrusion in very early developing spheroids

Our novel tool enabled the application of ECM-transmitted shear strain on breast spheroids. We further found a functional relationship between strain resistance and spheroid developmental status. In line with these findings, we next analyzed the potential impact of periodic shear strain on the development of breast cell spheroids by comparing strain responses of poorly developed (day 3–4) and low-developed (day 10–11) sample groups. For long-term cyclic strain experiments, a magnetic titanium mesh was used instead of a cover glass (for detailed information, see Supplementary Figure S1). The high porosity of the mesh with 20% open area guaranteed sufficient nutrition exchange between the media reservoir and the hydrogel-embedded cells. For cyclic stress application, the magnetic sled was moved cyclically from position -1 and position +1 (5 mm displacement each from the neutral position) to avoid gel creeping (cf. Figure 2D). Confocal images of the actin cytoskeleton were taken at the equatorial plane of embedded spheroids every 30 min for 22 h to monitor cell movement and deformation as direct strain reactions. The average applied strains were measured before (t = 0 h) and after cyclic stress application (t = 22 h) (Table 1).

**TABLE 1:** Quantification of cleaved caspase 3 (cC-3) positive spheroids and applied strain.

—	cC-3 positive spheroids	cC-3 negative spheroids	Proportion of cC-3 positive spheroids (%)	Average strain of spheroids 0 h (%)	Average strain of spheroids 22 h (%)
				Pos 0 → Pos 1	Pos 0 → Pos 1
Day 3 stressed	39	16	71	7.0	8.8
Day 3 control	8	46	14	—	—
Day 11 stressed	6	45	12	5.7	4.5
Day 11 control	8	54	13	—	—

**FIGURE 6:**
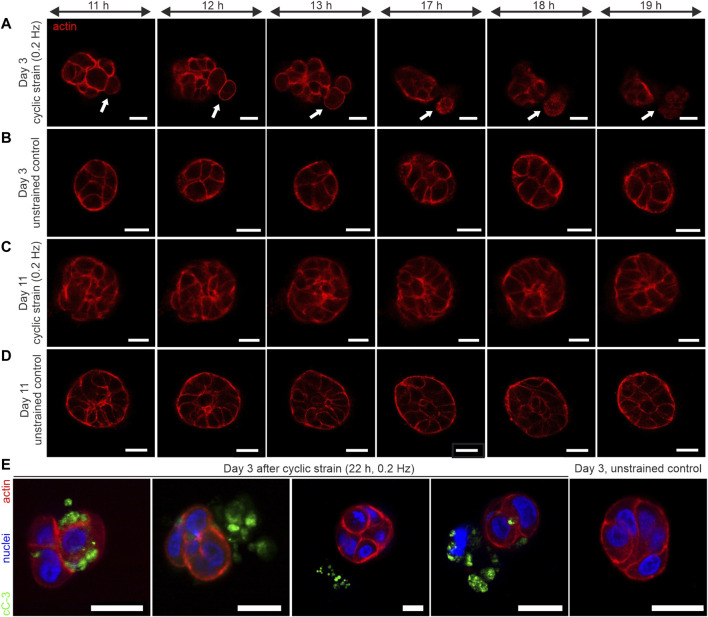
Long-term cyclic shear strain triggers cell extrusion and apoptosis in very early developmental stages of breast spheroids**.** MCF10A/RFP-LifeAct spheroids were cultivated for 3 and 11 days before being subjected to cyclic strain for 22 h (0.2 Hz, pos -1 and pos +1 = 5 mm). **(A)** The representative image series depicts the extrusion of 2 cells from a very early developed (day 3–4) spheroid body (white arrows). **(B)** Representative image series of a corresponding unstrained control sample. For complete image series of A and B, see Supplementary Movies S1, S2. **(C)** Image series of a representative strained spheroid (low-developed, day 10–11). **(D)** Representative image series of a corresponding unstrained control sample. **(E)** Shows four individual spheroids fixed and stained against cleaved Caspase-3 (cC-3) protein after 22 h of cyclic strain treatment. An unstrained control sample with the absence of cleaved caspase-3 is shown. All scale bars = 20 µm.

We tracked the actin cytoskeleton to capture changes in the general morphology of cells. The most striking result was that cyclic strain led to cell extrusion from the spheroid body. As shown in Figure 5A, rounding of cells within the cell cluster was observed (Supplementary Movie S1 and Figure 6A). This was followed by a subsequent detachment of single or pairs of cells from the cluster. Importantly, these cell extrusion events could only be observed in poorly developed spheroids (day 3 to day 4). In contrast, the unstrained control samples exhibited no changes in morphology over time (see Supplementary Movie S2 and Figure 5B).

On the contrary, based on the actin cytoskeleton, further developed spheroids (on day 10 to day 11) showed no cell rounding or extrusion events. These partially polarized spheroids (cf. Figure 5B) showed no shear strain-associated alterations, even after 22 h of mechanical actuation (Figure 6C, Supplementary Figure S1D). The corresponding unstrained control samples appeared similar to strained ones regarding their cytoskeletal shape and organization (Figure 6D; Supplementary Figure S1D). The actin cytoskeleton signal of extruded cells appeared structurally diffuse with fading intensity. This indicated the dying of cells during the extrusion process. Therefore, we tested for the presence of cleaved caspase-3 (cC-3) as a marker protein for executed apoptosis in spheroids after 22 h of cyclic strain. The immunostaining experiments confirmed the presence of cC-3 in spheroids that underwent cell extrusion. The results showed the presence of cC-3 within cells of the spheroid cluster. Further, the caspase-3 protein was present adjacent to cells in the ECM and frequently found next to fragmented nuclei, implicating that extruded cells underwent apoptosis. Unstrained spheroids were most often negative for cC-3 (Figure 6E).

To quantify the induction of strain-associated apoptosis, samples of strained and unstrained spheroids from three independent experiments were tested for cC-3, as shown in Figure 6E. The highest frequency of caspase-3 activation (71%) was found in poorly developed (day 4) spheroids. In contrast, the unstrained controls exhibited only occasionally apoptotic cells (14%). Interestingly, the frequency of apoptotic cells in low-matured spheroids (day 11) was similar to the unstrained control on day 4. In detail, only 12% of strained spheroids and 13% of unstrained ones showed caspase-3 activation (Table 1).

Together these results indicated that MCF10A breast spheroids in very early developmental stages are highly responsive to strain-induced apoptotic cell extrusion. A progressed differentiation, *i.e*., basoapical polarization and thicker basement membrane, protected from aberrant shear strain response that disturbed further spheroidal development.

## 4 Discussion

During its life cycle, breast gland tissue experiences a variety of ECM-transmitted stresses. For instance, milk-secreting luminal breast cells experience dynamic compressive stress exerted by contractile myoepithelial cells (Butcher et al., 2009). Deregulated mechanical cues foster basoapical polarization loss and finally, BM breaching and cell invasion (Whiteside, 2008; Chatterjee et al., 2012; Chang et al., 2017; Barbazán and Matic Vignjevic, 2019; Choi et al., 2019). Moreover, human breast gland tissue experiences substantial dynamic ECM-transmitted stresses caused by constant body movement, especially during exercise (Haake and Scurr, 2011). These mechanical cues generate dynamic and complex forces on the BM-covered epithelial breast cell lining. In contrast to fluid shear stress and uniaxial stress (Friedrich et al., 2019; Roux et al., 2020), ECM-transmitted solid shear stress remained poorly investigated as a fundamental regulator of cell function. This lack of knowledge was caused by missing experimental approaches to exert defined ECM shear strain on breast gland tissue.

Therefore, we created a magnetic shear stress device to investigate, for the first time, the mechanical stress response of a cell culture model of breast gland tissue. We designed our machine to apply solid shear strain on breast spheroids undergoing progressive basoapical polarization development. Three-dimensional morphogenesis of MCF10A spheroids is triggered only when cultured within their physiological niche, *i.e.*, an ultrasoft natural EHS-hydrogel. They never form on the surface of even soft cell culture substrates. We showed that our device could exert reproducible shear stress on such an ultrasoft matrix, which was crucial for all following cell analyses.

Further, we could prove that the hydrogel transmitted the cyclic shear stress to the spheroids. Our assay design kept optimal cell culture conditions during sample preparation and strain experiments. Hence, we did not observe unspecific side effects regarding gel properties or cell survival during sample preparation and analysis. Our setup enabled cyclic straining and real-time imaging of strained spheroids over extended periods. Cyclic straining of EHS-hydrogels and spheroids was tested for up to 22 h. Nevertheless, measurements over even more extended periods are technically feasible to analyze long-term adaptation. The ultimate limit of strain duration is set by aging or degradation of the matrix. The in-house developed image processing software enabled tracking structures formed by fluorescent marker beads in the matrix or by the actin cytoskeleton within the cells and thus measurement of local strain amplitudes within spheroids and the adjacent matrix. This dual tracking approach allowed for accurate strain calculation at each analyzed sample position. Further, to increase the number of acini analyzed, multiple positions within the sample could be analyzed sequentially within one sample.

We observed that at constant applied shear stress cell-conditioned hydrogels increased strain with prolonged cultivation time. This matrix softening argued for ongoing matrix remodeling by developing spheroids. In line with this, we and others have shown that MCF10A breast spheroids express matrix metalloproteases (MMP) under normal and tumorous ECM conditions (Yong et al., 2010; Eschenbruch et al., 2021). It is, thus, very likely that developing spheroids remodeled their physiological niche proteolytically. Therefore, MMP-driven remodeling of collagen type IV rich EHS-matrix could, in principle, be essential for the morphogenesis of MCF10A spheroids. However, less pronounced softening was also evident in native, cell-free gels. Previous work proved the presence of functional MMP-2, MMP-7, and MMP-9 in EHS-gels (Mackay et al., 1993; Gillette et al., 2003). Thus, gel softening is most likely the result of residual MMP activity, in which MMP-secretion of spheroids accelerated substantially. Overall, this finding underlines the sensitivity of our method by resolving even subtle stiffness changes in ultrasoft hydrogels. Future studies could use this feature to gain new insights into cell-modulated mechanical matrix remodeling.

Importantly, we found significant differences in strain between spheroids and their surrounding gel on day 11 and day 18 of cultivation. This finding indicated that spheroids acquired a mechanical strain resistance correlating with progressed maturation. Indeed, previous work demonstrated that the mechanical resistance against compressive forces of MCF10A spheroids increases significantly with BM-maturation (Gaiko-Shcherbak et al., 2015; Fabris et al., 2018). Hence, a maturation-dependent BM-strengthening very likely contributed to the observed shear stress resistance of breast spheroids. Possible underlying mechanisms are strain-induced changes in cell-cell junctions or cell-BM adhesions. However, clarification of the mechanisms is beyond the scope of this work. To confirm this direct relation of mechanical BM integrity and strain resistance, further analysis should compare the strain resistance of spheroids with or without enzymatic digestion of the BMs’ collagen IV network. Additionally, these studies should thoroughly examine the composition and localization of cell-cell and cell-matrix adhesions during the development of strained spheroids.

We further analyzed the cellular reaction of spheroids to long-term application of cyclic shear stress. Strikingly, we observed strain-induced extrusion of single or paired cells from the spheroid body. These cells undergo apoptotic cell death as indicated by cC-3 signal adjacent to spheroids. The poorly developed spheroids that showed this mechanoresponse experienced a cyclic shear strain of 8.8% at 0.2 Hz repeat frequency. Please note that these strain amplitudes are rather low compared to what breast tissue is experiencing during everyday life. For example, shear strain of 50% was measured in the adult female breast during exercise (Haake and Scurr, 2011). Nevertheless, on flat substrates, similar low strain regimes induce prominent cellular mechanoresponses. For instance, uniaxial strain (8%, 1 Hz) triggered actin cytoskeleton reorientation, modulation of pathways involved in cell-matrix adhesion and osteogenic differentiation in human mesenchymal stem cells (Zhang et al., 2021). Moreover, uniaxial strain (14%, 0.3 Hz) induced reorganization of mechanoresponsive focal adhesions and adherence junctions in differentiating epithelial skin cells (Noethel et al., 2018). Overall, we clearly showed that even such modest cyclic shear strain triggered significant mechanoresponse in BM-covered but polarization lacking spheroids (day 3—4). Particularly intriguing was that strain-induced apoptotic cell death and extrusion events occurred exclusively in the early stages of spheroid development on day 3 or 4 but not on day 11. Thus, the underlying transduction mechanisms of strain-induced cell extrusion and apoptosis are most likely functionally linked to BM maturation and apical polarization of the basal cell layer that takes place between day 3–4 and day 11 of development. Notably, maturation of BM scaffolds is accompanied by strengthening of the BM as indicated by its increased resistance to compressive force (Fabris et al., 2018). Thus, this strengthened structure could also shield the basal cell layer from ECM-transmitted strain (Gaiko-Shcherbak et al., 2015). The correspondingly lower local strain experienced by the cells themselves is most likely insufficient to induce strain-induced apoptotic cell extrusion. The observed caspase activation is accompanied by rounding of cells. This shape change also indicated the loss of cell-cell junctions, which could induce anoikis (Adeshakin et al., 2021).

While the underlying signaling circuits remain elusive, we propose the following scenario. We found that the basal cell layer of spheroids at day 3–4 formed short actin microspikes penetrating the thin BM. Moreover, it was shown that these ECM probing microspikes form focal adhesions to the BM and the ECM, especially in the early developmental stages of spheroids with low polarization (Eschenbruch et al., 2021). Considering actin-rich protrusions as the prevailing mechanism for ECM-mechanosensation, we hypothesize a shear strain-induced opening of PIEZO-1 ion channels as one of the primary sensing mechanisms. PIEZO-1 is a mechanosensitive ion channel that has been implied to drive mechanical signaling circuits in various cell types such as neuronal stem cells (Pathak et al., 2014), endothelial cells (Ranade et al., 2014) and keratinocytes (Holt et al., 2021). PIEZO-1 is expressed by MCF10A cells (Li et al., 2015). Current work suggests the tethering of PIEZO-1 to the actin cytoskeleton in mammalian epithelial MDCK cells (Wang et al., 2022). This should enable PIEZO-1 to sense the mechanical forces transmitted by F-actin. Additionally, apical cell extrusion from spheroids has been proposed to be linked to sphingosine phosphate signaling cascades (Gu et al., 2015), which depends on PIEZO-1 activation in epithelial colon cell monolayers and Zebrafish embryos (Eisenhoffer et al., 2012). Despite this growing body of evidence, further analysis is needed to confirm the involvement of PIEZO-1 in cell extrusion upon ECM-transmitted shear strain in developing breast gland spheroids. However, a thorough exploration of this hypothesis is beyond the scope of this work.

In conclusion, investigating cellular mechanoresponse and adaptation to ECM-transmitted solid shear strain has been an experimentally challenging task. The introduced device has unique features for applying cyclic shear strain on all types of cell cultures, from epithelial spheroids and primary organoids to neuronal networks. Its robust sample design enables easy adaptation for natural and synthetic ultrasoft hydrogels to resemble cell-specific matrix niches. The device is widely applicable and can be adapted to any inverted microscope. Using this device, we discovered an unknown mechanoresponse of human breast glad spheroids to ECM-transmitted shear strain. We are convinced that this approach will be of high value for studying ECM strain-mediated mechanobiological regulation circuits.

## Data Availability

The datasets supporting the conclusions of this article are either available within the paper and its Supplementary Material or from the corresponding author upon reasonable request.
