# Supplementary material for: ECM-transmitted shear stress induces apoptotic cell extrusion in early breast gland development
Source: Front Cell Dev Biol. 2022 Aug 29;10:947430. doi: 10.3389/fcell.2022.947430 (PMC9465044; doi:10.3389/fcell.2022.947430)
Supplement: Supplementary file 2 [file Presentation1.pdf]

Supplementary Information

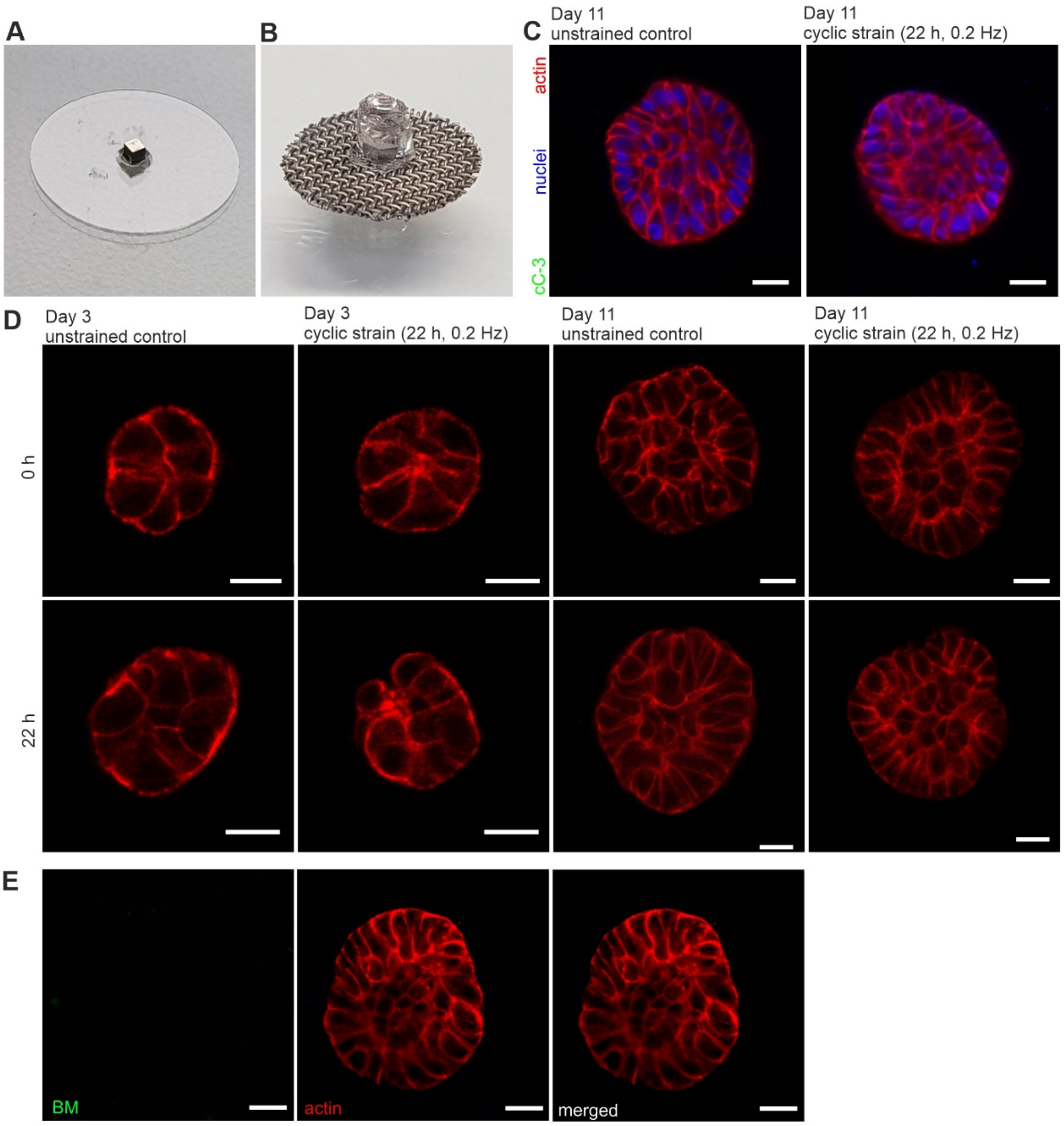

Figure S1: (A) A magnetic sample lid made with a cover glass without additional PDMS coating. **(B)** A magnetic sample lid made with a titanium mesh with 20% open surface allowing for higher molecular exchange between medium and hydrogel for shear stress application experiments. The magnet attached to the top of the mesh was covered in PDMS to avoid erosion of the magnet coating. **(C and D)** MCF10A/RFP-LifeAct spheroids were cultivated for 3 or 11 days before being subjected to cyclic strain (0.2 Hz for 22 hours, pos -1 and pos +1). **(C)** Representative images of strained and unstrained spheroids (day 11) fixed and stained against cleaved Caspase-3 (cC-3) protein. **(D)** Representative images of strained and unstrained spheroids before and after cyclic straining. Images at 0 h and 22 h for each condition show respectively the same spheroid. **(E)** Secondary antibody control for the

collagen tpe IV staining, showing no unspecific signal of the secondary antibody in MCF10A spheroids (day 11) (cf. Figure 5A). All scale bars = 20 µm.

Movie S1: MCF10A spheroid extrudes cells upon cyclic straining. MCF10A/RFP-LifeAct spheroids were cultivated for 3 days before being subjected to cyclic strain (0.2 Hz for 22 hours, pos -1 and pos +1). Images were acquired every 30 minutes for 22 hours. Top left counter indicates minutes past since start of the time series. Scale Bar: 50 µm.

Movie S2: Unstrained control MCF10A spheroid. MCF10A/RFP-LifeAct spheroids were cultivated for 3 days. Sample was prepared for shear strain application but was not strained. Images were acquired every 30 minutes for 22 hours. Top left counter indicates minutes past since start of the time series. Scale Bar: 50 µm.
